# Nutrients Profile of 52 Browse Species Found in Semi-Arid Areas of South Africa for Livestock Production: Effect of Harvesting Site

**DOI:** 10.3390/plants10102127

**Published:** 2021-10-07

**Authors:** Humbelani Silas Mudau, Hilda Kwena Mokoboki, Khuliso Emmanuel Ravhuhali, Zimbili Mkhize

**Affiliations:** 1 Department of Animal Science, School of Agricultural Sciences, Faculty of Natural and Agricultural Sciences, North West University, Mmabatho 2735, South Africa; hilda.mokoboki@nwu.ac.za; 2 Food Security and Safety Niche Area, Faculty of Natural and Agricultural Sciences, North-West University, Mmabatho 2735, South Africa; 3 Department of Chemistry, School of Physical and Chemical Sciences, Faculty of Natural and Agricultural Sciences, North West University, Mmabatho 2735, South Africa; zimbili.mkhize@nwu.ac.za

**Keywords:** browse species, chemical composition, amino acids, livestock, low quality forages

## Abstract

The use of these browse plant species as feed supplements to livestock is restricted due to a lack of knowledge about their nutritional status. This study was conducted to evaluate the nutritive value of woody browse species found in a semi-arid, as influenced by harvesting, site (Limpopo and North West Province). Limpopo had a Glenrosa, Mispah and Lithosols (GM-L) soil type and North West sites had an Aeolian Kalahari sand, Clovelly and Hutton (AKS-CH) soil type. Fresh leaves from fifty-two trees (five trees per species) were randomly selected and harvested from the site by hand-picking. Limpopo had forty-five browse species and North West had twenty-one browse species, respectively. The samples were air dried at room temperature and ground for laboratory analysis (nutritive value). The data were subjected to one-way analysis of variance (for those species that were not common in both sites) and two-way factorial (for those species that were common in both sites) in a completely randomized design. In the GM-L soil type, *M. azedarach* (223.2 g/kg DM) had the highest (*p* < 0.05) crude protein content (CP), whereas in the AKS-CH soil type, *V. hebeclada* (189.2 g/kg DM) had the highest (*p* < 0.05) CP content. Within each species, *V. nilotica. Subsp. Krasssiana* had the highest (*p* < 0.05) dry matter digestibility (725.4 g/kg DM), non-fibrous carbohydrates (607.3 g/kg DM), digestible energy (3.375 Mcal/kg) and metabolizable energy (2.771) content when compared to all the other browse species in both GM-L and AKS-CH soils. *Melia azedarach* in GM-L had the highest (*p* < 0.05) values in most amino acids’ parameters measured when compared to the same species in AKS-CH. Though the harvesting site had an effect on the nutritive value, all species, irrespective of the harvesting site, had sufficient CP to be used as a supplement to livestock exposed to the low-quality roughages. The results from this study will be useful for farmers and researchers through the provision of relevant information on how to improve livestock production. There is a need to run in vivo trials to determine the best species suitable for livestock sustainability.

## 1. Introduction

One of the main constraints to enhancing livestock productivity in Sub-Saharan region is a lack of feedstuff leading to poor nutrition. This is due to the fact that livestock mostly most rely on high fiber diets that have low nutrients such as protein, mineral, carbohydrates and vitamins. For livestock production in the Sub-Saharan areas, including South Africa, browse woody species have long been considered essential for livestock nutrition, particularly where the quantity (biomass) and quality of feedstuff is low for long periods [1,2]. Many authors highlight that during dry periods, these woody browse species can be used as an alternate feed to compensate for nutrient deficiencies and feed shortages [3,4,5,6], as most of them are semi deciduous during drought seasons when compared to grasses that become wilted [7,8,9], due to their higher nutrient content, specifically their crude protein (CP) levels, which normally range from 110 to 250 g/kg DM [1,10,11,12].

Spatial variation is also one of the factors influencing the concentration levels of nutrient elements in browse species [2,13]. Spatial variation involves several attributes such as area temperature [14,15], light- and ozone-influencing photosynthesis processes on plants, altitude [16], soil moisture, type and the fertility of certain areas [2,17,18,19]. 

The browse species are known to have bioactive compounds such tannins, phenols, flavonoids, alkaloids, saponins, polysaccharides, miscellanies organic acids, non-protein amino acids and other anti-nutritional factors, as demonstrated by Hassan et al. [20] and Ku-Vera et al. [21], that normally affect the nutrition and availability of nutrients such as protein and also hamper the degradability of substrates in the rumen, which result in a reduction in animal performance [22,23]. 

Evans and Messerschmidt [24] stressed that the significance portion of protein and amino acids is normally provided through the microbial biomass synthesis. Fiber fraction degradation and VFA production is promoted by microbial production for utilization by animals. Protein quality is when all essential amino acids (AAs) are available in correct proportions for a certain individual animal and vice versa [25] and a lack of a certain AA can affect animal productivity through a reduction in feed conversion efficiency due to nutritional deficiency [26]. 

Though there is a lot of information on the nutritive value of browse species worldwide, documentation on the assessment of the nutritive value of these browse species on a large scale of tree species from one region to another for the sustainability of livestock production is necessary. This information related to the nutritive value of these woody browse species distributed in semi-arid areas of South Africa can be useful to both communal and small-scale farmers. For the productivity of livestock, especially ruminants, in the rangelands, the level of concentration and other nutrients indicators should be established in order to locate these woody browse species in the ecological systems. A laboratory study is mandatory for a satisfactory understanding of the nutritional composition of these browse species dominating in the Savanna biome in arid and semi-arid communal areas. The information from the laboratory can assist farmers to distinguish the best common browse species with better nutritional properties. The objective of the current study was to determine the effect of the harvesting site (soil type) on the nutritive values of selected indigenous browse tree species, while hypothesizing that the harvesting site and plant species had an effect on the nutrient profile.

## 2. Results

The results of the chemical composition of the browse species found in GM-L soil are shown in Table 1. *Berchemia discolor* (216.2 g/kg DM) had the highest (*p* < 0.05) CP content when compared to *E. divinorum* (65.1 g/kg DM), which had the lowest (*p* < 0.05) CP content. *F. virosa* had the highest (*p* < 0.05) CF (67.2 g/kg), DMD (688.0 g/kg), DE (3.215 Mcal/kg) and ME (2.639 Mcal/kg) content when compared with *E. divinorum*, which had the lowest (*p* < 0.05) CF (13.5 g/kg), DMD (135.5 g/kg), DE (0.850 Mcal/kg) and ME (0.698 Mcal/kg) content. *Catha edulis* (463.1 g/kg DM) had the highest (*p* < 0.05) ADL content and *B. zeyheri* (499.3 g/kg DM) had the highest (*p* < 0.05) NFC content. 

In Table 2, for AKS-CH soil, the NFC content of *S. mellifera* (503.2 g/kg DM) was the highest (*p* < 0.05), while *P. velutina* (321.4 g/kg DM) and *V. erioloba* (315.2 g/kg DM) had the lowest (*p* < 0.05) NFC values. *Prosopis velutina* had the highest (*p* < 0.05) ash (93.3 g/kg DM) and CP (158.5 g/kg DM) content when compared to *S. lancea*, which had the lowest (*p* < 0.05) ash value (60.7 g/kg DM) and CP content of 83.2 g/kg DM. *S. mellifera* (50.3 g/kg DM) had the highest (*p* < 0.05) CF content.. *Vachellia erioloba* (300.6 g/kg DM) had the highest (*p* < 0.05) ADL content. *Diospros lycioides* had the highest (*p* < 0.05) DMD (661.2 g/kg DM), DE (3.100 Mcal/kg) and ME (2.545 Mcal/kg), while *V. erioloba* had the lowest (*p* < 0.05) DMD (442.8 g/kg DM), DE (2.165 Mcal/kg) and ME (1.778 Mcal/kg) content.

There were significant differences observed on the effect of species, soil type and the interaction between species and soil type on the measured parameters of browse species found in communal rangelands (Table 3). In GM-L, *M. azederach* (223.2 g/kg DM) had the highest (*p* < 0.05) CP content, whereas in AKS-CH, *V. hebeclada* (189.2 g/kg DM) had the highest (*p* < 0.05) CP content. *Schinus molle* had the highest (*p* < 0.05) CF and ADL content when compared to other browse species in GM-L. Within each species, with the exception of *P. africanum*, *S. molle*, *T. sericea*, *V. hebeclada* and *Z. mucronata*, all the other browse species had a higher (*p* < 0.05) ash content in GM-L when compared to the same species in AKS-CH. Within each species, *V. nilotica. Subsp. krasssiana* had the highest (*p* < 0.05) DMD (725.4 g/kg DM), NFC (607.3 g/kg DM), DE (3.375 Mcal/kg) and ME (2.771) content when compared to all the other browse species in both GM-L and AKS-CH soils.

The results of the effect of species and soil type on the soluble phenolics and condensed tannin of browse plant species in both soil types are presented in Table 4. There was a significant influence of browse species, soil type and browse species × soil type interaction on the soluble phenolics and condensed tannin concentration of browse leaves. The amount of soluble phenolics ranged from 0.0160 (*V. hebeclada*) to 0.1011% DM (*D. cinerea*) in the browse leaves harvested from GM-L, while the leaves from AKS-CH ranged between 0.0334 (*V. hebeclada*) to 0.1009% DM (*Z. mucronata*). *Vachellia hebeclada* leaves had the lowest (*p* < 0.05) condensed tannin concentration in both GM-L (0.70% DM) and AKS-CH (0.83% DM). Within each soil type (GM-L and AKS-CH), *P. africanum*, *T. sericea*, *V. hebeclada* and *Z. mucronata* had no significant difference in the condensed tannin concentration levels, while *V. tortilis* and *G. flava* had no significant difference in the soluble phenolic concentration levels.

The results of the effect of site and species on the amino acids’ profile of woody browse species are shown in Table 5, Table 6 and Table 7. Significance differences were observed in the effect of species, soil type and the interaction between species and soil type on the amino acids profile of the browse species found in communal rangelands. In GM-L and AKS-CH soil types, *S. molle* had the highest (*p* < 0.05) His content. In GM-L, *M. azederach* had the highest (*p* < 0.05) Arg (1.62 g/100 g), Ser (1.38 g/100 g), Gly (1.57 g/100 g), Asp (2.55 g/100 g), Glu (3.07 g/100 g), Thr (1.49 g/100 g), Ala (1.70 g/100 g), Lys (1.83 g/100 g), Met (0.26 g/100 g), Val (1.61 g/100 g), Ile (1.27 g/100 g) and Leu (2.35 g/100 g) content, as compared to the same species in AKS-CH.

## 3. Discussion

### 3.1. Chemical Composition of Browse Species

The nutritive content (Ash, CP, CF, ADL, NFC, DMD, DE and ME) in this study showed a wide significant variation among the browse species found in both soil types. These findings are consistent with Kraus et al. [14] and Ravhuhali et al. [2], who discovered that spatial variation has a great influence on the chemical composition of browse leaves. In this study, it was observed that the ash content from this study was higher in GM-L than AKS-CH, with a range between 35.7–136.7 g/kg DM. Ash concentration from this study was within the range (32.5–150.0 g/kg DM) reported by Aranga et al. [27] and Al Shafei and Nour [28]. The mineral matter known to be the inorganic matter in animals’ diet is regarded as ash [29], which can be a true reflection of the concentration levels of minerals (phosphorus, or potassium including a larger fraction of silica) in the plants. The results of the present study show that *Catha edullis* (463.1 g/kg DM) in GM-L and *V. erioloba* (300.6 g/kg DM) in AKS-CH had the highest ADL concentration in their respective areas. The highest lignin concentration tends to depress dry matter intake and digestibility, as demonstrated by Njidda et al. [30]. Boudet [31] stated that lignin is a component of the cell wall that is deposited and accumulated during the process of cell wall thickening. A high concentration of lignin is indigestible, and it reduces DM digestibility while increasing small particle outflow from the rumen [32]. The ADL results of the browse leaves in this study were within the range of 94.4–463.1 g/kg DM, and this was within the range of that reported by Njidda and Olatunji [33] and Al Shafei and Nour [28]. The differences in the amount of lignin in the browse plants in this study would normally be influenced by factors such as the plant species and harvesting site, which is in line with the results reported by Becerra-Moreno [34].

The harvesting site and browse species influenced CP concentration. All the browse species from AKS-CH sites had CP concentration ranges from 83.2 to 189.2 g/kg DM and these CP values are higher than the minimum protein requirement of 80 g/kg DM that is needed for optimal rumen microbial activities [35]. From this study, *M. azedarach* CP in GM-L (223.2 g/kg DM) was higher than that reported by Mokoboki et al. [36]. Mnisi and Mlambo [37] emphasized that browse species leaves mostly contain medium to high levels of CP content and their considered protein source for ruminants. The variation in CP concentration between species can be due to the inherent characteristics of each species, and the different location (altitude and soil type) related to the variation in protein content [1,38].

The dry matter digestibility results in the present study were ranging from 135.5–688.0 g/kg DM in GM-L and 442.8–725.4 g/kg DM in AKS-CH. The amount of acid detergent acid present in the substrates has a direct influence on the digestibility of the diets and most of the browse species in this study had higher DM digestibility values. These results can be influenceby different soil types and other environmental factors within various locations, as demonstrated by Kwaza et al. [39], Ravhuhali et al. [2] and Sariyidiz and Anderson [19]. Mlambo et al. [6] stated that cell wall composition, lignin and tannins are some of the elements that may influence the variance in digestibility among browse species.

The metabolizable energy results in this study ranged from 0.70–2.64 Mcal/kg in GM-L and 1.78–2.77 Mcal/kg in AKS-CH. All the browse species in the present study have an ME level within the recommended range (0.70–2.77 Mcal/kg) for the maintenance of ruminant production, especially goats [40]. Most browse plants from AKS-CH had a higher ME concentration when compared to GM-L on the same browse species. The ME results of the current study shows that these browse species have great potential to maintain and support high activities of livestock such as cattle and goats.

The digestible energy content in this study ranged from 0.85–3.22 Mcal/kg in GM-L and 2.17–3.38 Mcal/kg in AKS-CH. Digestible energy is a measurement of the amount of energy in a feed that is available for the animal to utilize. Non-Fiber Carbohydrate (NFC) is a mixture of starch, galactans, pectins, beta-glucans and simple sugars in various proportions. The NFC content in this study ranged from 18.4–532.1 g/kg DM in GM-L and 134.9–607.3 g/kg DM in AKS-CH. Parts of the NFC ferment more quickly than other portions in animal feed. Non-Fiber Carbohydrates can ferment very differently depending on their constituent source because of this non-uniformity, compromising rumen health.

Browse plant species are thought to channel their defensive compounds to nutritious plant parts because they are the most vulnerable to herbivores activities; however, herbivore exposure and the accessibility of browse leaves are also important factors determining phenolic distribution [7]. Bioactive compounds such as tannins and phenols have a negative impact in Sub-Saharan animal production, because most of the browse plants they feed on tend to accumulate high levels of these compounds. The phenolic concentration in the animal feedstuff may suppress feed intake and digestibility of the feed in which they are constituted. The results from the current study show that the browse species and the interaction between browse and the soil type (GM-L and AKS-CH) had an influence on the condensed tannins (CTs) and soluble phenolic content. From this study, *D. cinerea* (0.1011% DM), *Z. mucronata* (0.1009% DM) and *S. molle* (0.1000% DM) had the highest (*p* < 0.05) soluble phenolics concentration levels, which may cause a detrimental effect on animal health.

In this study, *D. cinerea* (222.58% DM) was much higher in the AKS-CH soil than the results reported by Ravhuhali et al. [41] on the same browse species in the same soil type. The concentration of CTs ranged from 0.70 (*V. hebeclada*) to 87.55% DM (*P. africanum*) in GM-L, and 0.83 (*V. hebeclada*) to 232.70% DM (*V. karroo*) in AKS-CH. The results of this study show that most of the browse species in AKS-CH had higher levels of tannin than those in the GM-L soil type.

Browse tree species have long been considered important for livestock nutrition, particularly where the quantity and quality of feedstuff are limited for prolonged periods [1,2,42]. In this study, variations were observed among species and harvesting sites and the interaction between species and harvesting sites. Variation in location, browse species and plant components are some of the interrelated factors that influence the forage nutritive value. These interrelated factors have been noted as determinants on the concentration levels of nutrient content and bioactive compounds in the browse species globally [2,13,43,44,45,46,47]. Spatial variation involves numerous attributes such as temperature, ozone, altitude, rainfall and different soil type [14,16,18].

Hasanuzzaman et al. [48] stated that variation in temperature is one of the environmental elements affecting plant growth and chemical composition in browse species. Temperature can produce several biochemical, physiological and molecular changes in the browse plant metabolism, including lipid liquefaction, protein denaturation and membrane integrity disruption, which influences secondary plant metabolites [49]. Results found by van Soets 43] indicated that when the temperature increases, there is an acceleration in the rate of cell lignification. The temperature effect was not a contributing factor from this study, as both sites had similar temperature levels.

Through observation in the study areas, both harvesting sites had different soil types, which might have influenced the outcome on the concentration level of nutrients in the plants. Sariyidiz and Anderson [19] emphasized that variation in soils, soil moisture and browse species and location had an influence on the chemical concentration levels of plants leaves. Kraus et al. [14] reported that soil serves as a growing medium for plants; however, plants grown on less fertile soil are expected to produce a high quantity of condensed tannins and other phenolics compounds. This might be opposite to these results due to the fact that most of the woody species depend on subsurface water rather than the topsoil that carries much of the nutrients. Plants grown on moderate to high fertile soil had lower levels of cellulose, lignin and acid detergent fiber when compared to low fertile soil that had higher cell lignification. According to Said-Al et al. [50], plants that are affected by drought stress tend to produce high levels of plant secondary metabolites in response to stress. Results reported by Ramakrishma and Ravishankar [17] indicate that during drought periods, the plants often activate oxidative stress, which increases the number of phenolic acids and flavonoids in the plants’ leaves as a protective function of the plants against drought stress. Becerra-Moreno [34] highlighted that water deficiency activates the increase in tannin polymerization of plant species, which tends to favor the accumulation of lignin. This is opposite to this study, which highlighted that species in areas that receive minimum rainfall (200 mm) such as Limpopo province (GM-L soil type) had a high concentration level of tannins when compared to North West Province (AKS-CH soil type) area that receives more rainfall (minimum, 400 mm).

Several reporters indicate that altitude tends to influence the spatial variation attributes such as soil fertility, soil moisture, temperature and sunlight [15,51,52]. Orwa et al. [53] reported that elevation had a huge impact on plant growth and chemical properties since it affects how much sunlight a plant receives, how many nutrients are accessible and how much water they can absorb in the soil. Results reported by Mountousis et al. [54] indicated that altitude had a vast substantial impact on plant leaves ash, crude fiber, crude protein and crude fat. According to Martz et al. [55], the amount of terpenoids and soluble phenolic compounds on plant leaves increased as altitude and latitude increased. These results indicate that the inductive mechanism influences the plant to produce certain chemical components and modify its chemical makeup in order to cope with different altitudes [16]. This is in agreement with the findings from this study whereby the browse species in a low altitude zone such as GM-L had lower phenolics and tannins when compared with the high altitude in AKS-CH. This lines-up with the results reported by Iriti and Faoro [15].

Yan et al. [56] highlighted that at high latitudes, plant nutrients normally have a higher concentration of nutrients on their leaves, which is the opposite to our findings. In this study, most tree leaves in lower latitude GM-L areas (22°40′21″ S, 22°28′35″ S and 23°08′10″ S) have higher nutrients concentrations when compared to those in higher latitudes (25°44′07″ S, 25°42′43″ S and 25°45′37″ S) in AKS-CH areas. Yan et al. [56] also highlighted that the strategy of nutrient allocation from low to high latitudes may be manipulated by temperature.

### 3.2. Amino Acids in Browse Species

Amino acids are crucial for all metabolic processes because they serve as building blocks for proteins and as intermediates in metabolism. The results of the present study show that browse species, soil type (except five parameters such as Gln, Phe, Met, Gly, Ser) and their interaction had an influence on the concentration of amino acids (AAs) in the browse leaves. In this study, *M. azedarach* in GM-L (1.61 g/100 g protein) had the highest valine content, which is likely to help with repairing damaged tissues, promoting normal growth and regulating blood levels [57]. Most of the browse species leaves in GM-L had higher AAs content when compared to the same species found in AKS-CH except in histidine, proline and tyrosine. Titgemeyer and Loest [58] stated that lysine, histidine, leucine, valine and methionine are limiting amino acids in livestock, especially cattle. With the linkages between dietary energy supply, amino acids supply and amino acids requirements, grazing cattle show strong performance responses to supplementary protein. Protein deposition can be thought of as an energy dependent process and, for the ruminal microbial protein synthesis, amino acid delivery is also an energy dependent. Ruminants fed grass silages may experience a limitation in amino acid supply and, thus, are capable of responding to supplementation with browse species. Various chemical compositions of different browse plant leaves, harvested from two different soil types (GM-L and AKS-CH) that have a similar temperature and with different altitudes and rainfall, are noted in Table 3, Table 4, Table 5, Table 6 and Table 7.

## 4. Materials and Methods

### 4.1. Description of the Harvesting Sites, Sampling and Laboratory Site

The harvesting sites were Thulamela Local Municipality and Makhado Local Municipality with a probably high number of livestock that completely rely on these communal rangelands. Table 8 provides information about the sampling sites. Limpopo had Glenrosa, Mispah and Lithosols (GM-L) soil type and North West sites had Aeolian Kalahari sand, Clovelly and Hutton (AKS-CH) soil type [59]. Different woody browse species were randomly chosen and then collected from two different Provinces of South Africa, namely Limpopo and North West Province. Fresh leaves (five trees per browse species) from fifty-two randomly selected trees species (*Adansonia digitate*, *Androstachys johnsoni*, *Balanites maughamii*, *Berchemia discolour*, *Berchemia zeyheri*, *Bridelia mollis hutch*, *Carissa edulis*, *Catha edulis*, *Colophospermum mopane*, *Combretum Imberbe*, *Combretum molle*, *Comretum collinum*, *Dalbergia melanoxylon*, *Dichrostachys cinerea*, *Diospros lycioides*, *Diospyros mespiliformis*, *Euclea divinorum*, *Flueggea virosa*, *Grewia flava*, *Grewia flavescens*, *Grewia monticola*, *Grewia occidentalis*, *Melia azedarach*, *Peltophorum africanum*, *Prosopis velutina*, *Pseudolachnostylis maprouneifolia*, *Pterocarpus rotundifolius*, *Schinus molle*, *Schotia brachypetala*, *Sclerocarya birrea*, *Searsia lancea*, *Searsia leptodictya*, *Searsia pyroides*, *Senegalia caffra*, *Senegalia galpinii*, *Senegalia mellifera*, *Senegalia nigrescens*, *Senegalia polyacantha*, *Strychnos madagascariensis*, *Terminalia sericea*, *Trichilia emetic*, *Vachellia erioloba*, *Vachellia hebeclada*, *Vachellia karoo*, *Vachellia nilotica*, *Vachellia nilotica subsp. Kraussiana*, *Vachellia rechmanniana*, *Vachellia robusta*, *Vachellia tortilis*, *Vachellia tortils subsp raddiana*, *Vangueria infausta*, *Ziziphus mucronata*) were harvested from the selected sites by hands-picking. Limpopo province had 45 browse species and North-West province had 21 browse species, respectively. Out of 45 species from Limpopo and 21 species from Northwest province, only 14 species were found to be common in both provinces. Each of the samples collected were stored in a brown paper bag per sample and labelled according to the scientific name of the tree species. The collected samples were air dried at room temperature for about seven days prior to grinding. The samples were ground to pass through a 1-mm sieve using a Wiley mill grinding machine and kept in tight plastic containers pending chemical analysis. Harvested browse species were also divided into groups based on their growth form, herbivory and preferred plant parts (Table 9a,b) [60]. Information not found in the textbook was obtained from the villagers or communal farmers.

The laboratory work was conducted at Molelwane experimental farm (25°48′00″ S and 25°38′21″ E) of North West University, Mafikeng campus. The experimental farm is situated 5.5 km west of North West University, South Africa.

### 4.2. Chemical Analysis

#### 4.2.1. Proximate Analysis

Approximately 1g of each woody browse leaves sample was weighed into pre-weighed crucibles and then incriminated in a muffle furnace set at 550 °C for about six hours (3–6 h). Ash was measured according to AOAC [61] method number 973.18. Acid detergent lignin (ADL) was determined by setting a dried acid detergent fiber (ADF) sample bag in 72% sulphuric acid (H_2_SO_4_) for 3 h to obtain an ADL value as demonstrated using Van Soets [62]. The total nitrogen (N) content was determined using the Kjeldahl as described using AOAC [63] method number 976.06. Afterwards was converted into crude protein (CP) by multiplying N content with a factor of 6.25. Solvent extraction procedure was used to determine crude total fat (CF) in animal feed as described using the AOAC Official Method 920.39 [64].

Non-fibrous Carbohydrates percentage (%NFC) was estimated using the following formula: %NFC=100−(% CF+% CP+% ASH+% NDF). DMD %=88.9−(0.779×% ADF), was the formula for dry matter digestibility. The following regression equation stated by Fonnesbeck et al. [65] was used to estimate digestible energy (DE, kcal/kg) using the dry matter digestibility values: DE (kcal/kg)=0.27+0.0428 (DMD %). DE values were converted to metabolizable energy (ME) using Khalil et al. [66] formula as follows: ME (Mcal/kg)=0.821 DE (Mcal/kg).

#### 4.2.2. Amino Acids

A water Acquity Ultra Performance Liquid Chromatograph (UPLC) with a photodiode Array (PDA) detector was used to separate and detect amino acids (AAs) as indicated by Ogbuewu et al. [67], Ananthan et al. [68] and Manyelo et al. [69]. One microliter (μL) of sample/standard solution was injected into the mobile phase, which transported the derivatized AAs to a 60 °C Water Ultra-Tax C 18 column (2.1 × 50 mm × 1.7 μm). A protein sample was first hydrolyzed (for example, with a strong acid) to release the amino acids, which are then extracted using chromatography, such as ion exchange, affinity or absorption. The analytes were eluted from the column by running a gradient. A Photodiode Array (PDA) detector was used to detect analytes eluting from the column, with each amino acid eluting at a different retention time.

### 4.3. Statistical Analysis

One-way analysis of variance under general linear model (GLM) procedure of SAS [70] was used to test the data on the effect of browse species on chemical composition for species that were not common in both harvesting sites. The following statistical model was used:

*Y_ij_* = *µ* + *B_i_* + *ε_ij_*

where *Y_ij_* is a dependent variable, *µ* is the overall mean, *B_i_* is the effect of browse species and *ε_ij_* is the error term associated with observation *ij*; the level of significance was set at *p* < 0.05.

Two-way analysis of variance under general linear model (GLM) procedure of SAS [70] was used to test the data on the effect of harvesting site/soil type and browse species on the chemical composition of 14 browse species common in both sites. The following statistical model was used:

*Y_ijk_* = *µ* + *B_i_* + *L_j_* + (*B* × *L*)*_ij_* + *ε_ijk_*

where *Y_ijk_* is a dependent variable, *µ* is the overall mean, *B_i_* is the effect of browse species, *L_j_* is the effect of two different soil types, (*B* × *L*)*_ij_* is the interaction effect between browse species and soil types and *ε_ijk_* is the error term associated with observation ijk and assumed to be normally and independently distributed. The method of least significant differences was set at *p* < 0.05 and was used to examine differences between means.

## 5. Conclusions

Though there was mixed variations, this study shows that different browse plant species, harvesting sites and the interaction between species and harvesting site have an effect on the nutritive value. All the browse plants harvested from both soil types (GM-L and AKS-CH) contained a medium to high protein content. These browse trees can be used as an alternative protein and energy supplement for ruminants during dry seasons when nutritive value of grass drops. The findings of this study will be useful for communal farmers, and local researchers in Sub-Saharan regions with relevant information on how to improve livestock production. Several studies are needed to determine the concentration levels of all the phytochemicals found in these browse species in order to improve and maximize the browsing of these woody species. There is also a need to run the in vivo trials to determine the best species suitable for livestock sustainability.

## Figures and Tables

**Table 1:** Chemical composition (g/kg DM, unless otherwise stated) of browse species found in GM-L soil type.

Species	Chemical Components							
	ASH	CP	CF	ADL	DMD	NFC	DE (Mcal/kg)	ME (Mcal/kg)
*A.digitata*	136.7^a^	168.7^f^	33.7^d^	174.6^q^	628.7^d^	167.6^r^	2.961^d^	2.431^d^
*A. johnsonii*	90.8^c^	69.3^u^	19.6^kl^	324.7^e^	399.3^v^	22.9^u^	1.979^v^	1.625^v^
*B. maughamii*	110.0^b^	133.4 ^k^	28.5^fg^	315.9^f^	415.0^u^	18.4^u^	2.046^u^	1.680^u^
*B. discolour*	63.8^efg^	216.2^a^	18.4^mn^	114.6^t^	638.0^b^	435.2^e^	3.001^b^	2.463^b^
*B. zeyheri*	63.9^efg^	129.8^l^	18.4^mn^	145.4^s^	63.442^c^	499.3^a^	2.985^c^	2.451^c^
*B. mollis H*	48.7^jklm^	98.8^p^	27.8^gh^	243.2^k^	554.4^i^	388.2^h^	2.643^i^	2.170^i^
*C. edulis*	54.9^ghijk^	82.0^t^	22.4^j^	273.1^h^	513.9^n^	176.3^q^	1.834^w^	2.027^n^
*Catha edulis*	76.8^d^	106.0^o^	15.5^p^	463.1^a^	365.5^w^	235.1^o^	2.469^n^	1.506^w^
*C. mopane*	46.5^klm^	124.7^m^	37.9^c^	265.4^i^	483.7^q^	331.9^l^	2.340^q^	1.921^q^
*C. imberbe*	59.6^fghi^	143.6^h^	23.7^i^	118.4^t^	610.7^e^	483.6^b^	2.884^e^	2.367^e^
*C. molle*	51.3^ijkl^	95.2^q^	17.8^mno^	352.5^d^	462.7^r^	262.7^n^	2.250^r^	1.848^r^
*C. collinum*	73.4^d^	129.7^l^	28.4^fg^	193.9^p^	614.1^e^	164.4^r^	2.898^e^	2.379^e^
*D. melanoxylon*	40.7^mn^	173.6^e^	18.7^lm^	326.6^e^	428.1^t^	175.5^q^	2.102^t^	1.726^t^
*D. mespiliformis*	103.9^b^	97.1^pq^	51.3^b^	304.1^g^	507.7^o^	81.6^t^	2.443^o^	2.005^o^
*E. divinorum*	44.5^lmn^	65.1^v^	13.5^q^	382.9^c^	135.5^z^	97.3^s^	0.850^z^	0.698^z^
*F. virosa*	74.1^d^	176.1^d^	67.2^a^	102.0^u^	688.0^a^	449.6^d^	3.215^a^	2.639^a^
*G. flavescens*	55.0^ghijk^	137.0^j^	20.0^k^	225.3^m^	512.4^n^	364.9^i^	2.463^n^	2.022^n^
*G. monticola*	53.1^hijkl^	116.8^n^	20.1^k^	312.2^f^	303.2^x^	187.9^p^	1.568^x^	1.287^x^
*G. occidentalis*	95.3^c^	157.8^g^	17.4^no^	162.6^r^	518.7^m^	241.3^o^	2.490^m^	2.044^m^
*P. maprouneifolia*	35.7^n^	86.1^s^	32.5^e^	175.8^q^	498.8^p^	408.5^f^	2.405^p^	1.974^p^
*P. rotundifolius*	57.5^ghij^	195.6^c^	17.0^o^	450.7^b^	273.6^y^	96.4^s^	1.441^y^	1.183^y^
*S. brachypetala*	44.8^lm^	116.0^n^	27.43^h^	216.5^n^	545.7^jk^	310.9^m^	2.605^jk^	2.139^jk^
*S. birrea subsp. caffra*	62.4^efgh^	143.4^h^	26.8^h^	252.4^j^	606.7^f^	241.7^o^	2.866^f^	2.353^f^
*S. nigrescens*	46.0^klm^	92.7^r^	15.3^p^	161.4^r^	444.8^s^	308.2^m^	2.174^s^	1.784^s^
*S. polyacantha*	55.4^ghijk^	200.6^b^	14.8^p^	246.6^k^	542.9^k^	343.3^k^	2.594^k^	2.129^k^
*S. madagascariensis*	39.8 ^mn^	92.6^r^	22.3^j^	173.0^q^	595.0^h^	464.1^c^	2.816^h^	2.312^h^
*T. emetic*	68.7^def^	140.4^i^	15.4^p^	236.7^l^	484.1^q^	354.4^j^	2.342^q^	1.923^q^
*V. nilotica*	45.3^lm^	116.5^n^	29.1^f^	263.3^i^	505.8^o^	398.7^g^	2.435^o^	1.999^o^
*V. rechmanniana*	46.2^klm^	92.1^r^	37.9^c^	263.6^i^	547.8^j^	324.8^l^	2.615^j^	2.147^j^
*V. tortils subsp. raddiana*	63.6^efg^	131.1^kl^	21.4^j^	211.5^o^	522.6^l^	363.0^i^	2.507^l^	2.058^l^
*V. infausta*	69.7^de^	131.9^kl^	22.0^j^	94.4^v^	599.7^g^	476.7^b^	2.837^g^	2.329^g^
SE	2.88	0.770	0.356	1.40	1.26	2.65	0.005	0.004

^a–z^ In a column, means with common superscripts do not differ (*p* > 0.05), Chemical components: CP: crude protein; CF: crude fat; ADL: acid detergent fiber; DMD: dry matter digestibility; NFC: non-fibrous carbohydrates; DE: digestible energy; ME: metabolizable energy; SE: Standard error; GM-L: Glenrosa, Mispah and Lithosols soil type.

**Table 2:** Chemical composition (g/kg DM, unless otherwise stated) of browse species found in AKS-CH soil types.

Species	Chemical Components							
	ASH	CP	CF	ADL	DMD	NFC	DE (Mcal/kg)	ME (Mcal/kg)
*D. lycioides*	87.7^b^	99.3^e^	36.3^c^	194.9^e^	661.2^a^	352.2^c^	3.100^a^	2.545^a^
*P. velutina*	93.3^a^	158.5^a^	33.8^e^	194.1^e^	565.1^d^	321.4^e^	2.689^d^	2.207^d^
*S. lancea*	60.7^d^	83.2^g^	28.8^g^	222.0^c^	600.1^c^	468.3^b^	2.838^c^	2.330^c^
*S. pyroides*	69.4^c^	114.8^d^	32.2^f^	207.7^d^	466.9^f^	335.8^d^	2.268^f^	1.862^f^
*S. mellifera*	68.9^c^	86.6^f^	50.3^a^	123.2^f^	644.8^b^	503.2^a^	3.030^b^	2.487^b^
*V. erioloba*	60.4^d^	129.1^b^	34.5^d^	300.6^a^	442.8^g^	315.2^e^	2.165^g^	1.778^g^
*V. robusta*	73.3^c^	125.7^c^	43.4^b^	230.7^b^	545.9^e^	346.4^c^	2.606^e^	2.140^e^
SE	1.48	0.466	0.130	1.46	1.25	2.19	0.0054	0.0044

^a–f^ In a column, means with common superscripts do not differ (*p* > 0.05), Chemical components: CP: crude protein; CF: crude fat; ADL: acid detergent fiber; DMD: dry matter digestibility; NFC: non-fibrous carbohydrates; DE: digestible energy; ME: metabolizable energy; SE: Standard error; AKS-CH: Aeolian Kalahari sand, Clovelly and Hutton soil type.

**Table 3:** Effect of species and soil type on chemical composition (g/kg DM, unless otherwise stated) of browse species found in GM-L and AKS-CH soil types.

Species	Chemical Components															
	ASH		CP		CF		ADL		DMD		NFC		DE (Mcal/kg)		ME (Mcal/kg)	
	GM-L	AKS-CH	GM-L	AKS-CH	GM-L	AKS-CH	GM-L	AKS-CH	GM-L	AKS-CH	GM-L	AKS-CH	GM-L	AKS-CH	GM-L	AKS-CH
*D. cinerea*	47.2^iA^	41.4^ghB^	119.4^jB^	137.5^fA^	12.5^iB^	21.0^iA^	283.8^bcA^	218.3^fgB^	510.1^jB^	571.4^eA^	199.5^jB^	451.5^bA^	2.453^jB^	2.716^eA^	2.014^jB^	2.230^eA^
*G. flava*	85.8^cA^	62.3^dB^	197.7^bA^	125.6^gB^	12.8^iB^	46.8^cA^	197.1^fgA^	186.7^hA^	492.6^lA^	492.1^kA^	182.3^kB^	352.2^gA^	2.378^lA^	2.376^kA^	1.953^lA^	1.951^kA^
*M. azedarach*	117.6^aA^	80.0^abB^	223.2^aA^	162.9^cB^	24.5^gA^	16.6^lB^	150.9^hA^	97.3^iB^	620.4^cB^	662.2^bA^	336.5^eB^	441.8^cA^	2.925^cB^	3.105^bA^	2.402^cB^	2.549^bA^
*P. africanum*	32.7^kB^	58.5^deA^	76.3^kB^	95.5^kA^	12.3^iB^	19.6^jA^	220.2^efB^	260.5^cdA^	646.7^bA^	597.0^cB^	280.1^hA^	134.9^lB^	3.084^bA^	2.825^cB^	2.494^bA^	2.320^cB^
*S. molle*	76.0^eA^	76.3^bA^	122.3^iA^	105.8^jB^	57.8^aB^	79.4^aA^	344.5^aA^	224.3^deB^	505.5^kB^	530.6^hA^	201.3^iB^	251.0^kA^	2.434^kB^	2.541^hA^	1.998^kB^	2.086^hA^
*S. leptodictya*	81.3^dA^	43.2^gB^	132.6^gA^	70.2^lB^	17.6^hB^	23.5^hA^	298.4^bA^	243.5^deB^	444.2^mB^	568.9^eA^	297.7^gB^	424.5^dA^	2.171^mB^	2.705^eA^	1.783^mB^	2.221^eA^
*S. caffra*	45.4^iA^	44.7^gA^	136.4^fB^	156.6^dA^	50.7^3bA^	41.3^bB^	274.6^cB^	316.9^aA^	561.7^eA^	493.7^kB^	367.7^bA^	292.0^iB^	2.674^eA^	2.383^kB^	2.195^eA^	1.957^kB^
*S. galpinii*	104.4^bA^	70.9^cB^	135.7^fA^	116.9^hB^	34.3^eA^	28.3^gB^	178.1^gB^	288.9^bA^	59.1.4^dA^	584.0^dB^	363.7^bcA^	247.6^kB^	2.801^dA^	2.770^dB^	2.300^dA^	2.274^dB^
*T. sericea*	37.6^jA^	36.6^iA^	78.2^kB^	180.8^bA^	26.4^fB^	34.6^eA^	223.1^deB^	275.1^bcA^	546.5^gB^	552.8^fA^	359.4^cdA^	294.1^iB^	2.609^gB^	2.636^fA^	2.142^gB^	2.164^fA^
*V. hebeclada*	59.1^fA^	56.5^efA^	157.2^dB^	189.2^aA^	43.5^cA^	35.5^dB^	226.7^deA^	219.2^fgA^	527.4^iA^	515.6^jB^	356.8^dA^	281.5^jB^	2.527^iA^	2.477^iB^	2.075^iA^	2.033^jB^
*V. karroo*	56.8^fgA^	53.2^fA^	175.5^cA^	108.0^iB^	24.8^gA^	14.0^mB^	244.1^dA^	186.9^hB^	536.5^hB^	541.8^gA^	319.4^fB^	405.0^cA^	2.566^hB^	2.589^gA^	2.107^hB^	2.125^gA^
*V. nilotica subsp. kraussiana*	54.5^ghA^	37.6^hiB^	152.0^eA^	137.5^fB^	11.5^jB^	30.1^fA^	182.8^gA^	84.9^iB^	509.1^jkB^	725.4^aA^	270.9^iB^	607.3^aA^	2.449^jkB^	3.375^aA^	2.011^jkB^	2.771^aA^
*V. tortilis*	84.3^cdA^	52.8^fB^	130.1^hA^	142.5^eA^	43.5^dA^	35.5^deB^	147.2^hB^	256.3^cdA^	553.1^fA^	475.6^lB^	334.3^eA^	313.1^hB^	2.637^fA^	2.306^lB^	2.165^fA^	1.893^lB^
*Z. mucronata*	51.8^hB^	82.3^aA^	131.3^ghA^	115.2^hB^	17.7^hA^	18.1^kA^	118.9^iB^	199.6^ghA^	657.4^aA^	525.2^iB^	532.1^aA^	399.9^fB^	3.084^aA^	2.518^iB^	2.532^aA^	2.067^iB^
SE	1.39		0.688		0.213		8.29		1.33		1.93		8.29		0.0047	

^a–m^ In a column, means with common lowercase superscripts do not differ (*p* > 0.05), ^AB^ In a row, means with common uppercase superscripts do not differ (*p* > 0.05), Chemical components: CP: crude protein; CF: crude fat; ADL: acid detergent fiber; DMD: dry matter digestibility; NFC: non-fibrous carbohydrates; DE: digestible energy; ME: metabolizable energy; SE: Standard error; GM-L: Glenrosa, Mispah and Lithosols soil type; AKS-CH: Aeolian Kalahari sand, Clovelly and Hutton soil type.

**Table 4:** Effect of species and soil type on soluble phenolics (SPh, % DM) and condensed tannin (CTs, % DM) of browse species found in GM-L and AKS-CH soil types.

Species	Soluble Phenolics		Condensed Tannins	
	GM-L	AKS-CH	GM-L	AKS-CH
*D. cinerea*	0.1011^aA^	0.0969^bB^	66.64^cdB^	222.58^bA^
*G. flava*	0.0801^eA^	0.0795^dA^	51.51^eB^	144.98^dA^
*M. azedarach*	0.0207^jB^	0.0830^cA^	1.14^iB^	46.22^gA^
*P. africanum*	0.0788^eA^	0.0758^eB^	87.55^aA^	87.46^fA^
*S. molle*	0.0377^iB^	0.1000^aA^	3.40^iB^	26.65^hA^
*S. leptodictya*	0.0360^iB^	0.0564^ghA^	2.43^iB^	114.34^eA^
*S. caffra*	0.0659^gA^	0.0514^iB^	71.31^cB^	165.42^cA^
*S. galpinii*	0.0869^dA^	0.0385^jB^	12.94^hA^	1.64^kB^
*T. sericea*	0.0908^cA^	0.0566^ghB^	80.88^bA^	84.61^fA^
*V. hebeclada*	0.0160^kB^	0.0334^kA^	0.70^iA^	0.83^kA^
*V. karroo*	0.0935^bA^	0.0582^fgB^	28.79^gB^	232.70^aA^
*V. nilotica subsp. kraussiana*	0.0897^cA^	0.0561^hB^	62.88^dA^	50.49^gB^
*V. tortilis*	0.0598^hA^	0.0598^fA^	35.30^fA^	7.37^jB^
*Z. mucronata*	0.0688^fB^	0.1009^aA^	15.90^hA^	18.02^iA^
SE	0.00066		1.65	

^a–k^ In a column, means with common lowercase superscripts do not differ (*p* > 0.05), ^AB^ In a row, means with common uppercase superscripts do not differ (*p* > 0.05), SE: Standard error; GM-L: Glenrosa, Mispah and Lithosols soil type; AKS-CH: Aeolian Kalahari sand, Clovelly and Hutton soil type.

**Table 5:** Effect of species and soil type on His, Arg, Ser, Gly, Asp and Glu (g/100 g sample) of browse species found in GM-L and AKS-CH soil types.

Species	His		Arg		Ser		Gly		Asp		Glu	
	GM-L	AKS-CH	GM-L	AKS-CH	GM-L	AKS-CH	GM-L	AKS-CH	GM-L	AKS-CH	GM-L	AKS-CH
*D. cinerea*	0.60^bA^	0.53^fB^	1.14^dB^	1.18^cA^	0.89^dA^	0.86^efB^	0.93^fB^	1.00^deA^	1.257^fA^	1.26^dA^	1.70^dA^	1.53^fB^
*G. flava*	0.50^cA^	0.24^hB^	1.31^cA^	0.78^iB^	0.87^dA^	0.60^iB^	0.99^eA^	0.70^iB^	2.210^bA^	1.30^dB^	1.91^bA^	1.50^fB^
*M. azedarach*	0.38^efB^	0.75^cA^	1.62^aA^	1.34^bB^	1.38^aA^	1.11^bB^	1.57^aA^	1.38^aB^	2.547^aA^	1.54^aA^	3.07^aA^	2.14^aB^
*P. africanum*	0.42^eB^	0.63^deA^	0.73^hB^	1.06^fgA^	0.58^hB^	0.90^deA^	0.66^iB^	0.97^efA^	0.870^iB^	1.28^dA^	1.16^iB^	1.66^cdA^
*S. molle*	0.72^aB^	0.93^aA^	1.16^dA^	1.09^efB^	0.97^cA^	0.94^cA^	1.20^bA^	1.00^deB^	1.540^cdA^	1.37^cB^	1.74^dA^	1.62^deB^
*S. leptodictya*	0.37^fA^	0.20^iB^	0.87^gA^	0.52^kB^	0.69^gA^	0.42^kB^	0.83^gA^	0.50^jB^	1.180^gA^	0.66^hB^	1.41^gA^	0.87^hB^
*S. caffra*	0.50^cB^	0.60^eA^	0.98^efB^	1.03^gA^	0.75^efB^	0.93^cdA^	0.91^fB^	1.01^dA^	1.007^hB^	1.21^eA^	1.53^fB^	1.62^deA^
*S. galpinii*	0.35^fgB^	0.67^dA^	0.97^efB^	1.13^deA^	0.73^fB^	0.80^hA^	0.78^hB^	0.95^fA^	1.380^eA^	0.97^fB^	1.64^eA^	1.37^gB^
*T. sericea*	0.33^gB^	0.39^gA^	0.58^iB^	0.70^jA^	0.36^iB^	0.51^jA^	0.48^jB^	0.67^iA^	0.830^iA^	0.71^gB^	0.86^jB^	0.92^hA^
*V. hebeclada*	0.46^dB^	0.79^bA^	1.00^eB^	1.40^aA^	0.89^dB^	1.21^aA^	0.97^eB^	1.30^bA^	1.340^eA^	1.37^cA^	1.69^deB^	2.15^aA^
*V. karroo*	0.72^aA^	0.64^dB^	1.57^bA^	1.21^cB^	1.06^bA^	0.88^efB^	1.21^bA^	1.11^cB^	1.293^fB^	1.37^cA^	1.93^bA^	1.58^eB^
*V. nilotica subsp. kraussiana*	0.59^bA^	0.54^fB^	1.27^cA^	1.16^dB^	0.95^cA^	0.85^fgA^	1.16^cA^	0.91^gB^	1.560^cA^	1.17^eB^	1.94^bA^	1.71^cB^
*V. tortilis*	0.52^cB^	0.56^fA^	0.94^fB^	1.08^fA^	0.77^eB^	1.10^bA^	0.85^gB^	1.09^cA^	0.970^hB^	1.46^bA^	1.35^hB^	1.77^bA^
*Z. mucronata*	0.73^aA^	0.55^fB^	1.16^dA^	0.98^hB^	0.97^cA^	0.83^ghB^	1.06^dA^	0.87^hB^	1.510^dA^	1.36^cB^	1.82^cA^	1.62^deB^
SE	0.014		0.015		0.014		0.013		0.016		0.018	

^a–k^ In a column, means with common lowercase superscripts do not differ (*p* > 0.05), ^AB^ In a row, means with common uppercase superscripts do not differ (*p* > 0.05), His: Histidine; Arg: Arginine; Ser: Serine; Gly: Glycine; Asp: Aspartic acid; Glu: Glutamic acid; SE: Standard error; GM-L: Glenrosa, Mispah and Lithosols soil type; AKS-CH: Aeolian Kalahari sand, Clovelly and Hutton soil type.

**Table 6:** Effect of species and soil type on Thr, Ala, Pro, Lys, Tyr and Met (g/100 g sample) of browse species found in GM-L and AKS-CH soil types.

Species	Thr		Ala		Pro		Lys		Tyr		Met	
	GM-L	AKS-CH	GM-L	AKS-CH	GM-L	AKS-CH	GM-L	AKS-CH	GM-L	AKS-CH	GM-L	AKS-CH
*D. cinerea*	0.87^dA^	0.84^eA^	0.84^efA^	0.82^efB^	1.39^cA^	1.08^dB^	0.82^dA^	0.70^deB^	0.89^fB^	1.02^dA^	0.10^eA^	0.08^ghiA^
*G. flava*	0.91^dA^	0.67^gB^	0.89^cdA^	0.80^fgB^	1.09^eA^	0.64^hB^	0.82^dB^	1.12^aA^	0.83^gA^	0.44^hB^	0.06^fA^	0.07^hiA^
*M. azedarach*	1.49^aA^	1.05^bB^	1.70^aA^	1.19^aB^	1.57^bB^	2.30^aA^	1.83^aA^	0.65^fgB^	1.11^cB^	1.23^aA^	0.26^aA^	0.25^aA^
*P. africanum*	0.55^hB^	0.87^deA^	0.58^iB^	0.83^defA^	0.61^jB^	0.79^fgA^	0.50^iB^	0.63^ghA^	0.49^jB^	0.85^fA^	0.06^fB^	0.16^cdA^
*S. molle*	0.96^cA^	0.95^cA^	0.86^deA^	0.77^gB^	0.81^hA^	0.75^gB^	0.49^iB^	0.56^jA^	1.13^bcA^	0.93^eB^	0.25^aA^	0.21^bB^
*S. leptodictya*	0.69^gA^	0.41^iB^	0.73^hA^	0.47^iB^	0.87^gA^	0.57^iB^	0.75^fA^	0.59^ijB^	0.58^iA^	0.37^iB^	0.14^bcdA^	0.05^ijB^
*S. caffra*	0.72^gB^	0.83^eA^	0.81^fgB^	0.86^cdeA^	0.88^gB^	1.01^eA^	0.80^deA^	0.56^jB^	0.95^eB^	1.07^cA^	0.14^bcdB^	0.18^bcA^
*S. galpinii*	0.77^fA^	0.74^fA^	0.78^gA^	0.69^hB^	0.76^iA^	0.80^fA^	0.96^bA^	0.38^kB^	0.76^hB^	1.01^dA^	0.14^bcdB^	0.19^bcA^
*T. sericea*	0.37^iB^	0.51^hA^	0.40^jB^	0.50^iA^	1.03^fA^	0.78^fgB^	0.40^jA^	0.35^kB^	0.41^kB^	0.62^gA^	0.03^fB^	0.09^fghA^
*V. hebeclada*	0.83^eB^	1.11^aA^	0.93^cB^	1.09^bA^	1.04^fB^	1.24^bA^	0.77^efB^	0.92^cA^	0.98^deB^	1.26^aA^	0.12^deA^	0.09^fghA^
*V. karroo*	1.02^bA^	0.84^eA^	1.01^bA^	0.87^cB^	1.64^aA^	1.05^dB^	0.89^cA^	0.68^efB^	1.25^aA^	1.15^bB^	0.17^bA^	0.12^efB^
*V. nilotica subsp. kraussiana*	0.97^cA^	0.86^deB^	1.00^bA^	0.88^cB^	1.17^dA^	1.00^eB^	0.98^bA^	0.97^bA^	1.15^bA^	0.84^fB^	0.13^cdeA^	0.02^jB^
*V. tortilis*	0.70^gB^	0.90^dA^	0.73^hB^	0.87^cdA^	0.82^hB^	1.16^cA^	0.67^gA^	0.61^hiB^	0.76^hB^	1.15^bA^	0.12^deA^	0.11^efgA^
*Z. mucronata*	0.99^bcA^	0.83^eB^	0.85^deA^	0.76^gB^	0.84^ghA^	0.76^fgB^	0.61^hB^	0.73^dA^	0.99^dA^	0.83^fB^	0.16^bcA^	0.13^deA^
SE	0.015		0.015		0.014		0.013		0.014		0.013	

^a–k^ In a column, means with common lowercase superscripts do not differ (*p* > 0.05), ^AB^ In a row, means with common uppercase superscripts do not differ (*p* > 0.05), Thr: Threonine; Ala: Alamine; Pro: Proline; Lys: Lysine HCl; Tyr: Tyrosine; Met: Methionine; SE: Standard error; GM-L: Glenrosa, Mispah and Lithosols soil type; AKS-CH: Aeolian Kalahari sand, Clovelly and Hutton soil type.

**Table 7:** Effect of species and soil type on Val, Ile, Leu, Phe and Gln (g/100 g sample) of browse species found in GM-L AKS-CH soil types.

Species	Val		Ile		Leu		Phe		Gln	
	GM-L	AKS-CH	GM-L	AKS-CH	GM-L	AKS-CH	GM-L	AKS-CH	GM-L	AKS-CH
*D. cinerea*	0.94^fB^	1.04^dA^	0.77^fgB^	0.85^cA^	1.37^dB^	1.44^dA^	1.29^deB^	1.40^eA^	0.15^bcA^	0.17^abA^
*G. flava*	0.98^eA^	0.78^jB^	0.76^fgA^	0.62^hB^	1.36^dA^	1.07^iB^	1.20^fA^	0.66^jB^	0.15^bcA^	0.10^dB^
*M. azedarach*	1.61^aA^	1.19^bB^	1.27^aA^	0.92^bB^	2.35^aA^	1.81^aB^	1.62^bB^	1.76^aA^	0.13^cdB^	0.17^abA^
*P. africanum*	0.67^hB^	0.96^efA^	0.54^iB^	0.78^deA^	0.95^gB^	1.39^eA^	0.68^iB^	1.08^gA^	0.11^dA^	0.13^cdA^
*S. molle*	1.07^dA^	0.90^ghB^	0.87^dA^	0.74^fB^	1.48^cA^	1.33^fB^	1.55^cA^	1.31^fB^	0.17^abA^	0.14^abcA^
*S. leptodictya*	0.84^gA^	0.50^lB^	0.69^hA^	0.41^jB^	1.19^fA^	0.70^kB^	0.85^hA^	0.53^kB^	0.13^cdA^	0.12^cdA^
*S. caffra*	0.91^fA^	0.86^iB^	0.74^gA^	0.68^gB^	1.35^dA^	1.31^fgA^	1.25^eB^	1.34^fA^	0.16^abcA^	0.17^abA^
*S. galpinii*	0.92^fA^	0.93^fgA^	0.78^fA^	0.79^dA^	1.26^eA^	1.27^ghA^	1.02^gB^	1.41^eA^	0.13^cdB^	0.17^abA^
*T. sericea*	0.41^iB^	0.55^kA^	0.33^jB^	0.45^iA^	0.59^hB^	0.84^jA^	0.50^jB^	0.82^iA^	0.13^cdA^	0.14^bcA^
*V. hebeclada*	0.92^fB^	1.30^aA^	0.77^fgB^	1.09^aA^	1.35^dB^	1.75^bA^	1.34^dB^	1.69^bA^	0.16^abcA^	0.17^abA^
*V. karroo*	1.30^bA^	1.12^cB^	1.06^bA^	0.93^bB^	1.74^bA^	1.54^cB^	1.78^aA^	1.57^cB^	0.19^aA^	0.18^aA^
*V. nilotica subsp. kraussiana*	1.18^cA^	0.97^eB^	1.00^cA^	0.77^defB^	1.71^bA^	1.41^deB^	1.65^bA^	1.03^hB^	0.17^abA^	0.14^bcA^
*V. tortilis*	0.84^gB^	1.01^dA^	0.70^hB^	0.85^cA^	1.22^efB^	1.39^eA^	1.03^gB^	1.48^dA^	0.15^bcA^	0.17^abA^
*Z. mucronata*	0.98^eA^	0.88^hiB^	0.82^eA^	0.75^efB^	1.45^cA^	1.24^hB^	1.33^dA^	1.10^gB^	0.14^bcdA^	0.13^cdA^
SE	0.014		0.013		0.016		0.014		0.013	

^a–l^ In a column, means with common lowercase superscripts do not differ (*p* > 0.05), ^AB^ In a row, means with common uppercase superscripts do not differ (*p* > 0.05), Val: Valine; Ile: Isoleucine; Leu: Leucine; Phe: Phenyalanine; Gln: Glutamine; SE: Standard error; GM-L: Glenrosa, Mispah and Lithosols soil type; AKS-CH: Aeolian Kalahari sand, Clovelly and Hutton soil type.

**Table 8:** Information on soil type, coordinates, altitude, distance and vegetation types of the sampling sites.

	Limpopo Province	North West Province
Harvested rangelands areas	Makuya, Mutele and Mpheni rangelands sites	Tsetse, Six hundred and Lepurong rangeland sites
Distance from each other	750 to 800 km from each other	
Municipality	Thulamela and Makhado Local Municipalities	Mahikeng and Ratlou Local Municipalities
Coordinates and altitude	Makuya (22°40′21″ S, 30°45′26″ E alt 639 m) Mutele (22°28′35″ S, 30°50′24″ E alt 339 m) Mpheni (23°08′10″ S, 30°03′18″ E alt 808 m)	Tsetse (25°44′07″ S, 25°39′40″ E alt 1296 m) Six hundred (25°42′43″ S, 25°37′32″ E alt 1300 m) Lepurong (25°45′37″ S, 24°59′54″ E alt 1162 m)
Soil type	Glenrosa, mispah and lithosols soil (GM-L)	Aeolian Kalahari sand, clovelly and hutton soil (AKS-CH)
Soil structure	Reddish or brown sandy to loamy soil	Clay-loamy to red brown sandy soil type
Temperature	13–34 °C	2–36 °C
Rainfall	200 to 500 mm	400 to 450 mm
Vegetation type	Soutpansberg Mountain Bushveld and makuleke sandy bushveld vegetations	Mafikeng Bushveld, Eastern Kalahari Bushveld and Thornveld vegetation

**Table 9:** Scientific and common names, growth form, herbivory and preferred plants of woody browse species located in semi-arid areas of two different provinces (Limpopo and North West) of South Africa.

(**a**)				
**Species**	**Common Name**	**Growth Form ^1^**	**Herbivores**	**Preferred Plants**
*A. digitata*	Boabab	T	Cattle, camel and game	Leaves, fruits and seeds
*A. johnsoni*	Lebombo-ironwood	T	Cattle, goats and game	Leaves and fruits
*B. maughamii*	Green thorn	T	Cattle, goats and game	Leaves, fruits and seeds
*B. discolour*	Brown ivory	T	Cattle, goats and game	Leaves, fruits and seeds
*B. zeyheri*	Red ivory	T	Cattle, goats and game	Leaves, fruits and seeds
*B. mollis hutch*	Velvet Sweet-berry	S	Cattle, goats and game	Leaves, fruits and twigs
*C. edulis*	Simple spined Num-num	S	Goats and game	Leaves and fruits
*C. edulis (Catha)*	Bushman’s tea	T	Cattle and goats	Leaves, fruits and seeds
*C. mopane*	Mopane	T	Cattle, goats and game	Leaves and pods
*C. Imberbe*	Leadwood	T	Cattle and goats	Leaves
*C. molle*	Velvet bush willow	T	Game	Leaves
*C. collinum*	Weeping bush willow	T	Cattle, goats and game	Leaves
*D. melanoxylon*	Zebra wood	S	Cattle, goats and game	Leaves and fruits
*D. cinerea*	Sekelbos/Sicklebus	S	Goats	Leaves
*D. lycioides*	Blue bush	S	Goats	Leaves, fruits and seeds
*D. mespiliformis*	Jackal berry	T	Cattle, goats and game	Leaves, fruits and seeds
*E. divinorum*	Magic guarri	S	Goats	Leaves, fruits and twigs
*F. virosa*	White berry-bush	S	Goats and game	Leaves and fruits
*G. flava*	Velvet raisin	S	Cattle, goats and game	Leaves, fruits and twigs
*G. flavescens*	Sandpaper raisin	S	Cattle, goats and game	Leaves, fruits and seeds
*G. monticola*	Silver raisin	S	Cattle, goats and game	Leaves and fruits
*G. occidentalis*	Cross berry	S	Cattle, goats and game	Leaves and fruits
*M. azedarach*	Seringa	T	Cattle and goats	Leaves
*P. africanum*	African-wattle	T	Cattle and goats	Leaves and pods
*P. velutina*	Velvet mesquite	S	Cattle, goats and game	Leaves, fruits and pods
*P. maprouneifolia*	Kudu berry	T	Goats and game	Leaves and fruits
(**b**)				
**Species**	**Common Name**	**Growth Form ^1^**	**Herbivores**	**Preferred Plants**
*P. rotundifolius*	Round-leaved blood wood	T	Cattle, goats and game	Leaves and twigs
*S. molle*	Peppertree	T	Cattle and goats	Leaves and fruits
*S. brachypetala*	Weeping boer-bean	T	Goats and game	Leaves, seeds and bark
*S. birrea*	Marula	T	Cattle, goats and game	Leaves, fruits and seeds
*S. lancea*	Karee	T	Cattle, goats and game	Leaves and fruits
*S. leptodictya*	Mountain karee	T	Cattle, goats and game	Leaves and fruits
*S. pyroides*	Common wild-currant	S	Cattle, goats and game	Leaves and fruits
*S. caffra*	Common hook thorn	T	Cattle, goats and game	Leaves and pods
*S. galpinii*	Monkey thorn	T	Goats and game	Leaves, pods and seeds
*S. mellifera*	Blackthorn	S	Cattle, goats and game	Leaves, pods and flower
*S. nigrescens*	Knob thorn	T	Cattle, goats and game	Leaves and pods
*S. polyacantha*	White-stemmed thorn	T	Cattle and goats	Leaves
*S. madagascariensis*	Black monkey-orange	S	Cattle, goats and game	Leaves and fruits
*T. sericea*	Silver cluster leaf	T	Cattle and goats	Leaves and gum
*T. emetic*	Christmas bells	T	Goats and game	Leaves, fruits and flower
*V. erioloba*	Camel thorn	T	Cattle, goats and game	Leaves and pods
*V. hebeclada*	Candle thorn	S	Goats and game	Leaves and pods
*V. karoo*	Sweet thorn	S	Cattle and goats	Leaves, pods and gum
*V. nilotica*	Scented-pod thorn	T	Goats and game	Leaves and pods
*V. nilotica subsp. Kraussiana*	Scented-pod thorn	T	Goats and game	Leaves and pods
*V. rechmanniana*	Silky thorn	T	Cattle, goats and game	Leaves and pods
*V. robusta*	Robust thorn	T	Goats and game	Leaves, pods and seeds
*V. tortilis*	Umbrella thorn	T	Cattle, goats and game	Leaves, pods and bark
*V. tortils subsp. raddiana*	Umbrella thorn	T	Cattle, goats and game	Leaves, pods and bark
*V. infausta*	Wild-medlar	S	Cattle, goats and game	Leaves and fruits
*Z. mucronata*	Buffalo-thorn	T	Cattle and goats	Leaves and pods

^1^ Growth form: T: tree; S: shrub.

## Data Availability

All data are available upon request.
